# Trends and practices following the 2016 hydroxychloroquine screening guidelines

**DOI:** 10.1038/s41598-023-42816-5

**Published:** 2023-09-20

**Authors:** Fritz Gerald P. Kalaw, Justin Arnett, Sally L. Baxter, Evan Walker, Brian Pedersen, Shyamanga Borooah

**Affiliations:** 1 Viterbi Family Department of Ophthalmology, Shiley Eye Institute, University of California San Diego, 9415, Campus Point Drive, La Jolla, CA 92093 USA; 2 Jacobs Retina Center, University of California San Diego, 9415, Campus Point Drive, La Jolla, CA 92093 USA; 3 Division of Ophthalmology Informatics and Data Science, Viterbi Family Department of Ophthalmology, 9415 Campus Point Drive, La Jolla, CA 92093 USA; 4 Health Department of Biomedical Informatics, University of California San Diego, 9500 Gilman Drive, La Jolla, CA 92093 USA; 5 Rheumatology, Allergy and Immunology, Department of Medicine, University of California San Diego, 9500 Gilman Drive, La Jolla, CA 92161 USA

**Keywords:** Drug safety, Toxicology, Medical imaging, Retinal diseases

## Abstract

This study aimed to understand the profile of hydroxychloroquine-treated patients, referral patterns, and dosing and to assess the adherence of eye care providers to the latest 2016 screening guidelines provided by the American Academy of Ophthalmology. Patients were identified using electronic health records (EHR) taking hydroxychloroquine and were seen by optometrists, retinal specialists, and non-retinal ophthalmologists. Review of EHR data includes demographic characteristics, indications, and dosing profile of hydroxychloroquine use, eye care provider managing the patient, and imaging modalities performed. A total of 166 patients were included in the study. The most common indications for screening were systemic lupus erythematosus and discoid lupus (52.4%) followed by rheumatoid arthritis (18.7%) and Sjögren’s syndrome (9.6%). Ninety-two (55.4%) patients were on a higher-than-recommended dose of > 5 mg/kg/day. Patients who weighed less (mean 63.9 kg) were taking a higher-than-recommended dose (vs. 81.5 kg, *p* < 0.001). Although retinal specialists adhered best to the use of all three recommended imaging modalities, visual field testing was done appropriately for only 8.3% of Asian and 71.1% of non-Asian patients. In conclusion, there is substantial variability in screening by ophthalmic providers and prescribing practices compared with the current recommendations. In particular, there is a marked deficiency in correct visual field testing in Asian patients. These findings are important to highlight potential interventions to improve screening for hydroxychloroquine toxicity.

## Introduction

Hydroxychloroquine (HCQ) is an immunomodulatory medication used to treat various autoimmune diseases. It received its United States Food and Drug Administration (FDA)-approval for treating systemic lupus erythematosus (SLE) in 1955^1, 2^. Although it is the first-line treatment for SLE, it has also been found useful in treating Sjögren’s Syndrome (SS), Rheumatoid Arthritis (RA), and a growing number of other indications^3–5^.

HCQ can cause retinal toxicity, a rare but well-recognized complication from its long-term use^6^. While the mechanism is not fully understood, it is known that HCQ is deposited in tissues that have a high melanin content, such as skin and the retinal pigment epithelium (RPE)^7^. In vitro studies indicate that HCQ alters the RPE lysosome pH, resulting in higher lipofuscin levels, a byproduct of photoreceptor degeneration and, eventually, RPE and photoreceptor loss^8^. As such, HCQ accumulation may lead to irreversible changes to retinal architecture and vision, and progression has been noted even after discontinuation^9^. Patients are usually unaware of early changes resulting from toxicity, and for this reason, ophthalmic screening of patients on HCQ is important^10^.

In 2002, the American Academy of Ophthalmology (AAO) released its first recommendation on screening for chloroquine (CQ) and HCQ retinopathy^11^. With further evidence and widespread use of newer imaging modalities, these guidelines were revised in 2011^12^, and later in 2016^13^. Current updated guidelines recommend that HCQ dosage be limited to ≤ 5 mg/kg/day of real body weight (a change from the 2011 standard which used ideal body weight); that patients receive baseline HCQ retinopathy screening within the first year of initiating therapy and then annually following 5 years of HCQ use, except for those with high-risk associations including renal disease, prior macular disease, concurrent tamoxifen use, for whom annual screening is recommended upon initiation; and that patients are screened with a dilated fundus examination, macular spectral-domain optical coherence tomography (SD-OCT), fundus autofluorescence (FAF) imaging, and automated Humphrey Visual Field (HVF) testing using the 10–2 paradigm. The exception was for patients of Asian ethnicity, for whom updated guidelines recommended using 24–2 or 30–2 visual field testing due to evidence of peripheral macular HCQ retinopathy in this cohort of patients^13^.

Despite evidence-based screening guidelines and known toxicity risks, compliance with HCQ retinopathy screening and appropriate HCQ dosage remains an issue. Papers have previously investigated rheumatologists, ophthalmologists, and dermatologists’ compliance with the guidelines provided by the AAO^14–19^. A survey by Sulman et al.^14^ in 2017 showed that only 5% of rheumatologists and 15% of ophthalmologists were aware of the recommended ophthalmologic assessments for baseline and follow-up examinations. Browning^17^ and Semmer et al.^15^ noted an increased screening frequency in low-risk patients within the first 5 years of therapy, indicating overutilized healthcare resources. Alternatively, Nika et al.^16^ noted a lack of routine monitoring for high-risk patients. Furthermore, Braslow et al. 9/15/2023 8:31:00 AM ^19^noted that greater than 50% of patients on HCQ therapy were on higher-than-recommended doses (> 5 mg/kg/day).

Our retrospective chart review aimed to investigate the prescribing patterns of HCQ of referring physicians and the screening practices of eye care providers in a large academic referral center based on the current 2016 guidelines.

## Methods

This study was approved by the Institutional Review Board of the University of California, San Diego (UCSD). The patients provided written informed consent as per institution protocol, and data collection and analysis were conducted according to the Principles of the Declaration of Helsinki. This study complied with the Health Insurance Portability and Accountability Act (HIPAA) of 1996.

### Data source and patient selection

Analysis of electronic health record (EHR) data available at the UCSD database was performed to identify records of patients included for retrospective review from January 1, 2016 until March 31, 2022. Patients were initially identified using structured queries of the EHR data warehouse (Epic SlicerDicer, Verona, WI, USA), including “Hydroxychloroquine sulfate” ever listed as an outpatient medication and patient encounter at the UCSD Viterbi Family Department of Ophthalmology. Patients noted in the eye care provider’s chart as a screening for HCQ were included. Eligibility was confirmed with each patient via manual chart review.

### Chart review

Chart review of the EHR system included review of ocular examination data, encounter notes, and ophthalmic testing data and images indexed on the Zeiss Forum software version 4.2.4.15 (Zeiss, Oberkochen, Germany) application. Variables recorded from the chart review included self-reported gender, self-reported race, indication for HCQ therapy, age at starting HCQ therapy, date of first HCQ screening, the type of eye care provider conducting the screening exams (categorized as optometrist, retinal specialist, or non-retinal ophthalmologist), daily dose at the first screening, weight (in kg), highest daily HCQ dose, date of last screening, total duration of HCQ therapy, and if the patient was still taking HCQ at the time of chart review (April 2022). Further information, collected from the patient data, included concurrent tamoxifen use, known renal disease, prior retinal or ophthalmic disease, systemic comorbidities, as well as the modalities used to screen for HCQ retinopathy, including HVF (10–2 and 24–2), SD-OCT, FAF, and multifocal electroretinogram (mfERG). The patient data were collected in a de-identified, encrypted database.

### Statistical analysis

Data normality was initially assessed through the Shapiro–Wilk testing. Subject characteristics were presented as count (%) and mean (95% CI) for categorical and continuous variables, respectively. Demographic tables present *p*-values derived from t-tests, Fisher’s Exact test, and Chi-Squared tests for subject-level characteristics. Demographic tables consisting of a comparison of more than two cohorts present *p* values derived from analysis of variance (ANOVA), Fisher’s Exact test, and Chi-Squared tests for subject-level characteristics. A further analysis was conducted utilizing linear mixed-effects modeling to account for within-group variability of subjects attending the same type of eye care provider. A post hoc analysis was implemented to assess the significance of differences between pairs of group means using Tukey’s honestly significant difference (HSD) test with degrees of freedom estimated by Satterthwaite’s approximation. Mean mg/kg/day ratios were estimated for each indication of HCQ therapy, and a one-tailed t-test was implemented to evaluate if ratios were significantly greater than a mu of 5 mg/kg/day. The statistical analysis was done using the R Statistical Programming Software (Version 4.2.1). A *p* value less than or equal to 0.05 were considered statistically significant.

## Results

The data from the EHR initially yielded 287 patients, of which 166 were noted to have eye care provider’s clinic notes on HCQ screening upon visit; hence, were included in the study. Table 1 shows the demographic characteristics of the patients included in the study. Ninety percent of patients were female and 48.8% were white. The mean duration of HCQ therapy was 5.3 years and the mean estimated daily dose was 5.2 mg/kg. The majority of the patients underwent SD-OCT, FAF, and HVF. None of the patients were noted to have signs of HCQ retinopathy.

**Table 1:** Demographics of patients seen for hydroxychloroquine screening.

	Patients on HCQ (n = 166)
Sex	
Female (%)	150 (90.4%)
Male (%)	16 (9.6%)
Race	
Asian (%)	24 (14.5%)
Black or African American (%)	7 (4.2%)
Other or mixed race (%)	50 (30.1%)
Unknown (%)	4 (2.4%)
White (%)	81 (48.8%)
Estimated age starting HCQ in years	
Mean (CI)	44.2 (41.7, 46.8)
Weight in kg	
Mean (CI)	71.7 (68.9, 74.6)
Duration of therapy in years	
Mean (CI)	5.3 (4.4, 6.1)
Estimated daily dose in mg/kg	
Mean (CI)	5.2 (5.0, 5.5)
Cumulative dose in grams	
Mean (CI)	684.4 (568.5, 800.4)
High-risk^a^	
No (%)	110 (66.7%)
Yes (%)	55 (33.3%)
SD-OCT screen	
No (%)	7 (4.2%)
Yes (%)	159 (95.8%)
FAF screen	
No (%)	18 (10.8%)
Yes (%)	148 (89.2%)
HVF screen	
No (%)	47 (28.3%)
Yes (%)	119 (71.7%)

*HCQ* Hydroxychloroquine, *SD-OCT* Spectral-domain optical coherence tomography, *FAF* Fundus autofluorescence, *HVF* Humphrey visual field.

^a^High-risk features including renal disease, concomitant tamoxifen use, known retinal or macular disease.

We examined characteristics leading to higher-than-recommended HCQ dosage (Table 2). Of the 166 patients that were reviewed, 92 (55.4%) had daily doses greater than the AAO-recommended 5 mg/kg/day with a mean cumulative lifetime dose of 783.6 g as opposed to the mean cumulative lifetime dose of 561.2 g in the ≤ 5 mg/kg/day cohort (*p* = 0.06). Those at higher-than-recommended dosages were, on average, at a lower weight than those at appropriate dosage (a mean of 63.9 kg vs. 81.5 kg, respectively, *p* < 0.001). No statistically significant differences were observed in the likelihood of higher-than-recommended dosage based on the indication for HCQ therapy, duration of therapy, high-risk profile, and the type of eye care provider performing the screening.

**Table 2:** Extended differences between patients that received doses above and below 5 mg/kg.

	≤ 5 mg/kg (n = 74)	> 5 mg/kg (n = 92)	*p* value
Weight in kg (mean [CI])	81.5 (76.5, 86.5)	63.9 (61.8, 66.0)	** < 0.001**^a^
Duration of therapy in years (mean [CI])	5.0 (3.9, 6.2)	5.5 (4.2, 6.7)	0.594
Cumulative lifetime dose in grams (mean [CI])	561.2 (429.2, 693.1)	783.6 (603.5, 963.6)	0.060
Indication for HCQ therapy (total [%])			
1. UCTD, MCTD	3 (4.1%)	5 (5.4%)	0.711
2. RA	13 (17.6%)	18 (19.6%)	
3. SLE, Discoid lupus	41 (55.4%)	46 (50.0%)	
4. Inflammatory arthritis, uncertain	3 (4.1%)	8 (8.7%)	
5. Sjögren	9 (12.2%)	7 (7.6%)	
6. Other^b^	1 (1.4%)	4 (4.3%)	
7. Multiple^c^	4 (5.4%)	4 (4.3%)	
Attending Group (% group)			
Ophthalmologist	18 (24.3%)	19 (20.7%)	0.695
Optometry	15 (20.3%)	24 (26.1%)	
Retina	41 (55.4%)	49 (53.3%)	
High-Risk^d^ (% group)			
No	43 (58.9%)	67 (72.8%)	0.069
Yes	30 (41.1%)	25 (27.2%)	

*HCQ* Hydroxychloroquine, *UCTD* Undifferentiated connective tissue disease, *MCTD* Mixed connective tissue disease, *RA* Rheumatoid arthritis, *SLE* Systemic lupus erythematosus.

^a^Bold *p*-values are less than 0.05.

^b^Other diseases involved: Lichen planus, Dermatomyositis, Palindromic rheumatism, Unspecified dermatitis, Lichen sclerosis, Systemic sclerosis, Osteoarthritis, Autoimmune neutropenia, Psoriatic arthritis.

^c^Multiple autoimmune diseases involved: SLE + Dermatomyositis, Sjögren + Scleroderma, SLE + Sjögren, SLE + RA, RA + Sjögren, SLE + Scleroderma, RA + Sjögren + Lichen sclerosis, SLE + Juvenile idiopathic arthritis, SLE + Psoriatic arthritis.

^d^High-risk features including renal disease, concomitant tamoxifen use, known retinal or macular disease.

**Figure 1:**
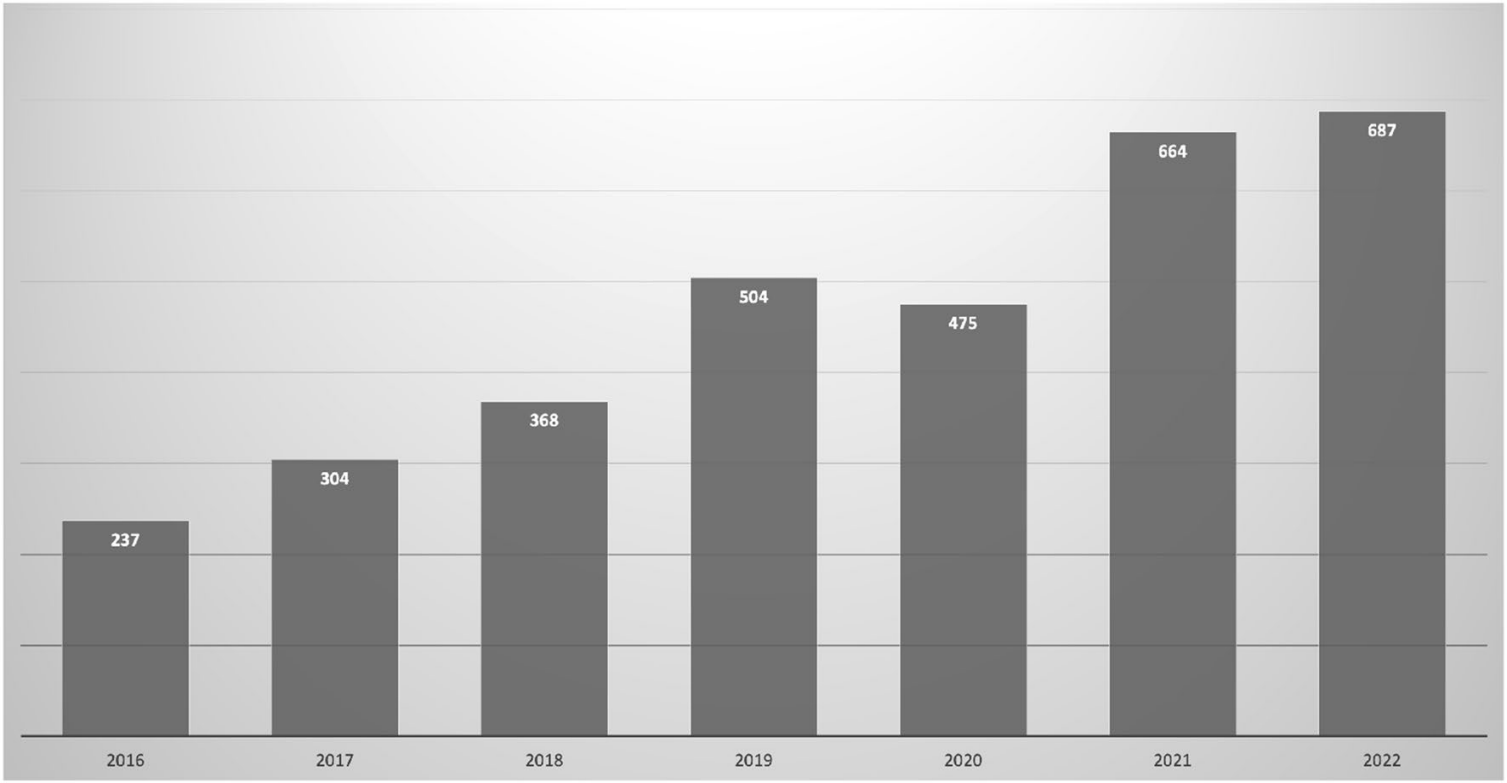
Annual visits of patients taking hydroxychloroquine sulfate seen at our eye institution.

We characterized individual indications for HCQ therapy as seen in Table 3. The most common indications for HCQ use were SLE and discoid lupus (87/166 [52.4%]), followed by RA (31/166 [18.7%]) and Sjögren’s Syndrome (16/166 [9.6%]). Interestingly, there was an increasingly diverse range of conditions being treated with HCQ in the other category including lichen planus, dermatomyositis, dermatitis, lichen sclerosis, systemic sclerosis, osteoarthritis, autoimmune neutropenia and psoriatic arthritis. There was a progressive increase in HCQ retinopathy screening annually (Fig. 1). Taken together, this suggests that the number of patients prescribed and conditions treated with HCQ is increasing, therefore increasing the risk of an increase in absolute numbers of HCQ toxicity cases.

**Table 3:** Differences between indications for hydroxychloroquine therapy.

	UCTD, MCTD (n = 8)	RA (n = 31)	SLE, Discoid lupus (n = 87)	Inflammatory arthritis, uncertain (n = 11)	Sjögren (n = 16)	Other^b^ (n = 5)	Multiple^c^ (n = 8)	*p* value
Estimated age starting HCQ (mean [CI])	54.5 (39.7, 69.3)	58.9 (54.3, 63.6)	36.1 (33.3, 38.9)	50.5 (39.2, 61.9)	50.3 (41.1, 59.5)	40.2 (20.5, 59.9)	46.9 (36.2, 57.5)	** < 0.001**^a^
Duration of therapy (mean [CI])	5.4 (2.0, 8.7)	4.1 (2.8, 5.5)	6.3 (4.9, 7.8)	3.5 (1.1, 5.9)	4.2 (2.7, 5.6)	3.4 (− 0.2, 7.0)	3.8 (1.1, 6.5)	0.288
Cumulative lifetime dose (mean [CI])	684.4 (162.7, 1206.0)	541.6 (350.3, 733.0)	835.2 (638.3, 1032.0)	484.5 (146.8, 822.1)	438.0 (302.7, 573.3)	408.8 (− 42.6, 860.2)	538.4 (133.6, 943.1)	0.239

*HCQ* Hydroxychloroquine, *UCTD* Undifferentiated connective tissue disease, *MCTD* Mixed connective tissue disease, *RA* Rheumatoid arthritis, *SLE* Systemic lupus erythematosus.

^a^Bold *p* values are less than 0.05.

^b^Other diseases involved: Lichen planus, Dermatomyositis, Palindromic rheumatism, Unspecified dermatitis, Lichen sclerosis, Systemic sclerosis, Osteoarthritis, Autoimmune neutropenia, Psoriatic arthritis.

^c^Multiple autoimmune diseases involved: SLE + Dermatomyositis, Sjögren + Scleroderma, SLE + Sjögren, SLE + RA, RA + Sjögren, SLE + Scleroderma, RA + Sjögren + Lichen sclerosis, SLE + Juvenile idiopathic arthritis, SLE + Psoriatic arthritis.

The age at which HCQ therapy was started was different among the disease groups (*p* < 0.001), as the SLE group started on average the earliest (36.1 years), and the RA group started on average the latest (58.9 years). The SLE group had the longest mean duration of therapy (6.3 years) and the highest cumulative dose (835.2 g) on initial screening. However, there was no statistically significant difference among the comparison of all cohorts.

We examined differences in patient characteristics and screening practices based on eye care provider type, focusing on retinal specialists, non-retinal ophthalmologists, and optometrists, as seen in Table 4. There was no statistically significant difference between patient characteristics seen by the different eye care providers in terms of duration of therapy, cumulative dose, and high-risk profile, suggesting that the groups saw similar patients. Optometrists were less likely to employ FAF (vs. ophthalmologists [*p* = 0.019], retina [*p* < 0.001]) and ophthalmologists were less likely to employ HVF (vs. optometrist [*p* = 0.031], retina [*p* = 0.016]) on screening visit when compared to their counterparts (Table 5). Of note, only 2/24 (8.3%) Asian patients underwent the recommended 24–2 test pattern and only 101/142 (71.1%) non-Asian patients underwent the recommended 10–2 test pattern. Baseline mfERG, which is an optional test, was performed on only 5 patients by a single retinal specialist. One-hundred fifty-eight (158/166) patients underwent at least two structural and functional screening modalities (HVF, SD-OCT, FAF), the most common of which was the aforementioned three modalities (105/158), followed by SD-OCT and FAF (41/158), then SD-OCT and HVF (11/158). Only one patient underwent simultaneous FAF and HVF without SD-OCT.

**Table 4:** Extended differences between patients seen by each eye care providers.

	Ophthalmology (n = 37)	Optometry (n = 39)	Retina (n = 90)	*p* value
Duration of therapy in years				0.634
Mean (CI)	5.2 (3.0, 7.4)	6.0 (3.9, 8.1)	5.0 (4.0, 5.9)	
Cumulative dose in grams				0.668
Mean (CI)	697.4 (376.0, 1018.9)	771.2 (523.2, 1019.2)	641.5 (504.0, 778.9)	
High-risk^b^				0.296
No	23 (62.2%)	30 (76.9%)	57 (64.0%)	
Yes	14 (37.8%)	9 (23.1%)	32 (36.0%)	
SD-OCT screen				0.121
No	1 (2.7%)	4 (10.3%)	2 (2.2%)	
Yes	36 (97.3%)	35 (89.7%)	88 (97.8%)	
FAF screen				** < 0.001**^**a**^
No	2 (5.4%)	13 (33.3%)	3 (3.3%)	
Yes	35 (94.6%)	26 (66.7%)	87 (96.7%)	
HVF screen				**0.010**^**a**^
No	18 (48.6%)	8 (20.5%)	21 (23.3%)	
Yes	19 (51.4%)	31 (79.5%)	69 (76.7%)	

*SD-OCT* Spectral-domain optical coherence tomography, *FAF* Fundus autofluorescence, *HVF* Humphrey visual field.

^a^Bold *p* values are less than 0.05.

^b^High-risk features including renal disease, concomitant tamoxifen use, known retinal or macular disease.

**Table 5:** Pairwise comparison between imaging modalities performed by eye care providers.

	SD-OCT	FAF	HVF
General ophthalmology versus Optometry	0.431	**0.019**^**a**^	**0.031**^**a**^
General ophthalmology versus Retina	0.986	0.851	**0.016**^**a**^
Optometry versus Retina	0.164	** < 0.001**^**a**^	0.934

*SD-OCT* Spectral-domain optical coherence tomography, *FAF* Fundus autofluorescence, *HVF* Humphrey visual field.

^a^Bold *p* values are less than 0.05.

## Discussion

Several FDA-approved oral, topical, and intravitreal medications have been demonstrated to cause toxicity to the retina^20^. For over two decades, HCQ toxicity has been carefully scrutinized, and evidence-based data documenting the toxicity have led to continually updating screening guidelines. However, despite the different disciplines' efforts to provide care for patients on HCQ, screening remains poor. Our study explored the patient and physician characteristics leading to differences in HCQ screening practices since the 2016 AAO screening guidelines. A survey by Winebrake et al.^21^ in 2020 regarding the perspective of rheumatologists on the 2016 AAO guidelines cited that limited dosing options, lack of supporting evidence, and low patient adherence were the main obstacles to implementing the guidelines. However, in 2020, the American College of Rheumatology, American Academy of Dermatology, Rheumatologic Dermatology Society, and the American Academy of Ophthalmology provided a joint statement on HCQ use with respect to retinal toxicity^22^.

The most common indications for HCQ therapy screening in our cohort were SLE and RA, consistent with a previous 2013 study^17^. Other indications for HCQ use were also seen in our study, which comprised 30.7% (51/166) of our cohort. Several researchers investigated screening and retinal toxicity identified in patients with SLE and RA^2, 5, 16, 23–27^. However, screening for HCQ retinopathy from indications other than SLE and RA has not been carefully studied. In our academic center, we found that the rate of patients prescribed with HCQ from all indications appeared to be increasing annually. This will likely lead to an increase in the number of patients at risk of toxicity if screening and dosing are not performed appropriately. Awareness of the use of HCQ, aside from SLE and RA, should warrant eye care providers of a more comprehensive assessment to identify changes for possible HCQ retinopathy, even though the clinic visit was not primarily set up to address HCQ screening.

Six years following the presentation of the most updated AAO screening guidelines, adherence to the HCQ dosing guidelines remains a major concern. Our group observed that 55.4% of the patients in our cohort were taking HCQ at a daily dose greater than the recommended 5 mg/kg/day, a finding similar to a previous 2017 study (56%), based on the 2011 AAO screening guidelines^19^. The patients in our cohort were likelier to weigh less than their counterparts at a ≤ 5 mg/kg/day recommended dosage, and the mode daily dose in this cohort was 400 mg. As new guidelines have shifted towards a basis of real (or actual) weight^13^, some patients on higher-than-recommended dosages may still have been taking the medication based on the prior-recommended basis of ideal body weight^12^. As of 2022, smaller incremental dosages (100 mg, 200 mg, 300 mg, and 400 mg) of HCQ are available, which may provide more options of more optimized dosing. Another way to overcome higher-than-recommended dosing was suggested in a study by Chen et al.^28^ that analyzed the impact of an EHR-based clinical decision support (CDS) tool by alerting the prescribing clinician of an HCQ dose greater than 5 mg/kg of actual body weight or 400 mg daily dose, whichever is lower. The study noted a significant decrease in the prevalence of excessive dosing based on the 2016 AAO guidelines.

Although cumulative dose had been thought to be a strong indicator of risk, the new AAO guidelines cited that the risk is more accurately assessed based on the duration of use relative to daily per-weight dosage^13^. If HCQ is taken at 4.0–5.0 mg/kg/day, the 15-years cumulative risk of retinopathy is around 5–7.5% but can be as high as 20–25% if taken at > 5.0 mg/kg/day^29^. This means that, in our cohort, 55.4% of patients are at risk for developing HCQ retinopathy if they continue to use the higher-than-recommended dose.

Regarding eye care providers’ adherence to the latest guidelines, we observed that retinal specialists were most compliant in screening their patients, including screening with all three recommended modalities. This increased compliance is likely related to a keener awareness of retinal specialists to the updated AAO recommendations and further dedication of retinal specialists to HCQ screening during the patient encounter. However, a deficiency was adhering to the recommended test patterns for Asians (24–2) for all eye care providers. A study by Kim, et al. noted a higher sensitivity of the 30–2 (90.6%) test pattern in detecting early pericentral retinopathy compared to 10–2 (53.1%) test pattern and a higher specificity of the 10–2 (89.6%) test pattern compared to 30–2 (84.8%) test pattern in detecting overall retinopathy among Asian patients taking HCQ^30^. This shows an important use of a broader test pattern to detect early changes within the peripheral macular areas in the Asian population.

There are a few limitations to our study. Given the retrospective nature of this study, records obtained from the electronic medical system may generate inaccuracies regarding the different data collected particularly for the cumulative dose of HCQ. However, as described previously, based on updated guidelines, the duration of therapy relative to the per-weight dose was a more accurate risk prediction; thus, identifying the initiation of treatment would provide less error than computing the total regimen dose. Also, since this was a single-institution study, our conclusions are less generalizable.

Based on our data, our group recommends and suggests the following with respect to HCQ retinopathy screening: 1. Increasing awareness of existing and upcoming screening guidelines not only for eye care providers but also for the HCQ-prescribing clinicians; 2. Increasing awareness to eye care providers that diseases other than SLE and RA might be treated with HCQ and thus require screening; 3. Highlight that Asian patients should be screened using 24–2 or 30–2 HVF rather than using the standard 10–2 testing. 4. Using EHR resources (such as a CDS tool) to flag that high-risk medications, such as HCQ or tamoxifen, are being prescribed to the eye care provider’s patient, to avoid missed screening and to promote discussion for non-toxic use as well as implementation to alert prescribing clinicians of a possible toxic dose of > 5 mg/kg/day may be helpful; 5. Developing streamlined eye care providers or HCQ-prescribing clinician’s order sets to facilitate compliant screening; 6. Communication and collaboration between physicians to prevent under- or overtreatment; and 7. Consideration for prescribers to use tablets of all currently available dosage forms (100 mg, 200 mg, 300 mg, and 400 mg) to facilitate physician prescribing per guidelines.

In conclusion, adherence to the updated screening guidelines remains a concern. Our study demonstrated that there is still room for improvement in adhering to the current AAO guidelines for HCQ retinopathy screening in an academic setting, as the trends and practices elucidated in the present study highlight specific deficiencies and potential initiatives for improvement. Proper dosing and imaging modalities should be reviewed to manage patients with chronic HCQ therapy carefully. Eye care providers should be aware of the updated criteria, particularly using the suggested wider visual testing patterns when screening Asian patients.

## Data Availability

All data in the current study are available from the corresponding author (SB) upon reasonable request.
